# Steel Slag-Enhanced Cement-Stabilized Recycled Aggregate Bases: Mechanical Performance and PINN-Based Sulfate Diffusion Prediction

**DOI:** 10.3390/ma19030546

**Published:** 2026-01-29

**Authors:** Guodong Zeng, Hao Li, Yuyuan Deng, Xuancang Wang, Yang Fang, Haoxiang Liu

**Affiliations:** 1 Foshan Transportation Science and Technology Co., Ltd., Foshan 528041, China; wutongshuxi_a@163.com (G.Z.); hao_li_20007@163.com (H.L.); dfy8307@163.com (Y.F.); 2 School of Highway, Chang’an University, Xi’an 710064, China; yuyuandeng@chd.edu.cn (Y.D.); lhxpro@126.com (H.L.)

**Keywords:** pavement base, cement-stabilized recycled aggregate, steel slag aggregate, mechanical performance, sulfate diffusion, sulfate resistance, physics-informed neural network model

## Abstract

The application of cement-stabilized recycled aggregate (CSR) in pavement bases is constrained by the high porosity and low strength of recycled aggregate (RA), whereas sulfate transport and durability mechanisms are less reported. To address this issue, this study incorporated high-strength and potentially reactive steel slag aggregate (SSA) into CSR to develop steel slag-enhanced cement-stabilized recycled aggregate (CSRS). The mechanical performance of the mixtures was evaluated through unconfined compressive strength (UCS) and indirect tensile strength (ITS) tests, and their durability was assessed using thermal shrinkage and sulfate resistance tests. In addition, a sulfate prediction model based on a physics-informed neural network (PINN) was developed. The results showed that, compared with CSR, the 7-day and 28-day UCS of CSRS increased by 6.7% and 16.0%, respectively, and the ITS increased by 4.3% and 5.9%. Thermal shrinkage tests indicated that CSR and CSRS, incorporating RA and SSA, exhibited slightly higher thermal shrinkage strain than cement-stabilized natural aggregate (CSN). During sulfate attack, SSA significantly improved the sulfate resistance of CSR, with the sulfate resistance coefficient of CSRS increasing by 18.8% compared to CSR. Furthermore, the PINN model predicted that, in 3%, 5%, and 7% sodium sulfate solutions, the sulfate concentration at a 1 mm depth in CSRS was reduced by 35.6%, 21.8%, and 29.4%, respectively, compared to CSR, with an average relative error below 14%, confirming its reliability. Therefore, these findings demonstrate that the incorporation of SSA markedly enhances the mechanical properties and sulfate resistance of CSR, and that the PINN model provides an effective tool for accurate simulation and prediction of sulfate diffusion.

## 1. Introduction

With the ongoing global urbanization and the continuous expansion of infrastructure construction, the generation of construction and demolition (C&D) waste has increased markedly and has become one of the main sources of solid waste, typically accounting for 30~40% of the total solid waste output [1,2]. Waste concrete constitutes the largest proportion of C&D waste and represents the most critical component requiring urgent valorization. Through crushing, screening, and separation, waste concrete can be processed into RA, enabling the reduction, recycling, and environmentally sound disposal of C&D waste [3,4].

Numerous studies have shown that RA has broad application potential in pavement engineering. When used to partially replace natural aggregate (NA) in cement-stabilized base materials, RA can effectively reduce construction costs and mitigate the increasing scarcity of natural aggregate resources [5,6]. Nevertheless, RA typically retains porous residual mortar and contains internal microcracks, leading to high porosity, high water absorption, and insufficient strength. Consequently, its mechanical performance and durability remain inferior to those of NA [7,8]. Shi et al. [9] reported that the incorporation of RA into concrete pavements leads to reductions in elastic modulus and splitting tensile strength, ultimately degrading the overall mechanical performance. Similarly, Sadati et al. [10] reported that incorporating 30% to 40% RA markedly reduced the compressive strength and elastic modulus of concrete, indicating reductions in stiffness and load-bearing capacity. Medina et al. [11] found that when RA replaced 25% and 50% of NA in concrete, its workability was not affected, whereas the density, compressive strength, and durability decreased as the replacement ratio increased. The high porosity and water absorption of RA lead to marked deterioration in concrete performance and structural integrity, which has substantially limited its broader application. Therefore, a practical and effective approach is urgently needed to improve the overall performance of RA-incorporated concrete and enhance its engineering applicability.

In recent years, the application of steel slag aggregate (SSA) in pavement materials has attracted increasing attention [12,13,14,15]. Owing to its high hardness and angular particle morphology, SSA can significantly enhance the strength and stiffness of cement-stabilized base mixtures. Appropriately proportioned SSA contributes to a denser aggregate skeleton and improved particle interlocking [16,17,18]. Several reviews have summarized the benefits of SSA in cementitious materials, highlighting its potential to improve mechanical performance, durability, and sustainability [19,20]. Cai et al. [21] showed that in ultra-high-performance geopolymer concrete with steel slag, a 9.3–13.8% steel slag content optimized compressive strength, elastic modulus, and flexural performance. Zhao et al. [22] investigated alkali-activated fiber reinforced composites with steel slag partially replacing fly ash and ground granulated blast-furnace slag. With 15% steel slag replacement, the composites achieved a tensile strength of 4.86 MPa, tensile strain capacity of 3.94%, and compressive strength of 93 MPa.

These benefits are further confirmed by experiments on cement-stabilized mixtures showing improved strength and stiffness with SSA. Chen et al. [23] observed that the 28-day UCS of cement–stabilized steel slag (CSS) increased by 23.68% compared with CSN when SSA, naturally aged for one year, was used as a complete replacement for NA. Liu et al. [13] found that the UCS of cement-stabilized base mixtures increased with the SSA content, with the mixture containing 50% SSA exhibiting the highest UCS. However, untreated SSA may contain free CaO, which can induce expansion, reduce strength, and increase the risk of swelling failure [23,24]. Other studies have evaluated SSA in cement-stabilized bases regarding mechanical properties, volume stability, and environmental impact, confirming its practical applicability in road base materials [25,26,27].

Moreover, the incorporation of SSA has demonstrated excellent resistance to sulfate attack. Zhang et al. [28] reported that modified steel slag concrete maintained a low Ca^2+^ concentration in the leachate after 10 days of accelerated sulfate exposure, indicating reduced formation of corrosion products and enhanced material stability. Cheng et al. [29] found that replacing 60% of the coarse aggregate with SSA in a 15% sulfate environment increased compressive strength by 20.7%. In addition, Vo et al. [30] observed that concrete with full SSA replacement exhibited minimal mass change in sulfate solution, demonstrating great corrosion resistance. Additional studies have investigated long-term durability and lifetime prediction of SSA-containing mixtures, confirming its potential for enhancing sulfate resistance [29,31]. These findings highlight the potential of SSA for improving the sulfate resistance of CSR.

Furthermore, Steel slag, a major by–product of the steelmaking process, is generated in significant quantities, typically accounting for 15–20% of crude steel production [32,33]. The large-scale stockpiling of steel slag not only occupies land but also poses environmental risks [34,35,36]. Incorporating SSA into RA can enhance the utilization of steel slag and address the strength deficiencies of CSR. This approach not only maximizes the potential of two types of waste but also supports the development of a circular economy by promoting the resource recovery of construction waste [37].

However, current studies remain limited in several aspects. First, the effects of SSA incorporation on the mechanical and durability performance of CSR have not been sufficiently examined, particularly its resistance to sulfate attack. In addition, theoretical models capable of describing sulfate diffusion in CSR are still lacking, which hinders the accurate prediction of its sulfate resistance.

Previous studies have shown that sulfate attack, often coupled with freeze–thaw or wet–dry cycles, induces complex deterioration in concrete, involving ion transport, moisture movement, and microstructural changes [38,39,40]. Traditional experimental and modeling approaches often require large datasets or rely on simplifying assumptions, limiting prediction accuracy. To address these challenges, physics-informed neural networks (PINN), an advanced deep learning algorithm that integrates governing equations, have been increasingly applied to simulate sulfate diffusion in cementitious systems, enhancing the capabilities of conventional neural networks [41,42]. By integrating physical laws into engineering models, PINN can develop high-precision numerical simulations using only a small amount of data [43,44]. Shaban et al. [45] applied PINN to model chloride diffusion mechanisms and accurately predict chloride concentration distributions in concrete. These studies indicate that PINN can also be a promising tool for simulating sulfate diffusion in the CSR.

In this context, this study evaluated the mechanical and durability performance of CSR incorporating SSA through UCS, ITS, thermal shrinkage, and sulfate attack tests. A PINN-based diffusion–reaction model was developed to predict sulfate transport and to examine the effects of RA and SSA on the sulfate migration process. This work provides both experimental evidence and a predictive modeling tool to support the synergistic use of SSA and RA in pavement base construction.

## 2. Materials and Methods

### 2.1. Raw Materials

The cementitious material used in this study was ordinary Portland cement (OPC, PO 42.5), produced by Shaanxi Qinling Cement Co., Ltd., Tongchuan, China. Its basic properties were measured in accordance with the Chinese standard Test Methods of Cement and Concrete for Highway Engineering (JTG 3420-2020) [46], as shown in Table 1. Anhydrous sodium sulfate (Na_2_SO_4_) was purchased from Sinopharm Chemical Reagent Co., Ltd., Shanghai, China. Barium sulfate (BaSO_4_) was obtained from Tianjin Huasheng Chemical Reagent Co., Ltd., Tianjin, China.

The aggregates used in this study included recycled aggregate (RA), steel slag aggregate (SSA), and natural aggregate (NA). The RA was obtained from demolished concrete pavement and was crushed and screened into two size ranges (5–10 mm and 10–20 mm). The 20–30 mm fraction was retained as NA to ensure adequate strength, and its basic properties were measured according to the Technical Specifications for the Utilization of Construction Waste in Highway Engineering (JTG/T 2321–2021) [47].

The SSA was sourced from hot-stewed basic oxygen furnace steel slag that had weathered for one year. Its water-immersion expansion rate, determined following Test Method for Stability of Steel Slag (GB/T 24175–2009) [48], was 1.76%. Its basic properties were measured in accordance with Steel Slag for Road (GB/T 25824–2010) [49]. The NA was limestone, and its basic properties were tested following the Test Methods of Aggregates for Highway Engineering (JTG 3432–2024) [50]. The basic properties of RA, SSA, and NA are summarized in Table 2.

### 2.2. Specimen Preparation

**Figure 1:**
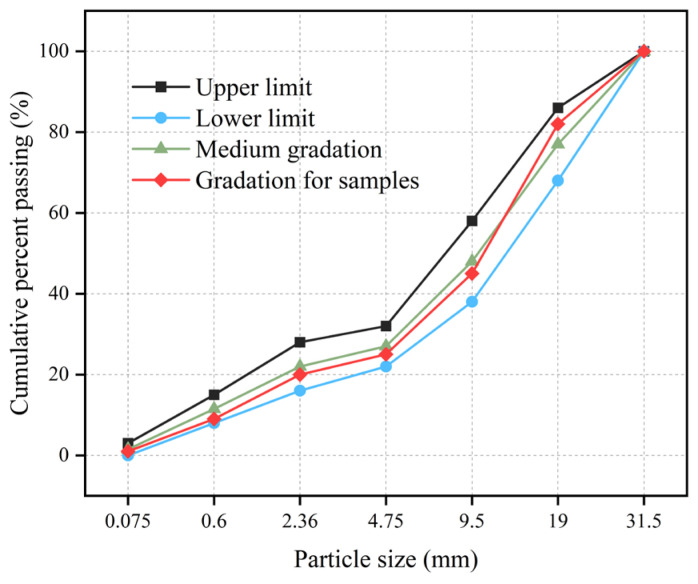
Gradation curve of cement-stabilized materials.

The gradation curve of the cement-stabilized material was designed following the Technical Guidelines for Construction of Highway Roadbases (JTG/T F20-2015) [51], as shown in Figure 1. Cylindrical specimens with a diameter of 150 mm and height of 150 mm, as well as beam specimens measuring 100 × 100 × 400 mm, were prepared following the Test Methods of Materials Stabilized with Inorganic Binders for Highway Engineering (JTG 3441-2024) [52]. Based on previous findings [53], in which a D-optimal mixture design was used to identify the optimal replacement ratios, RA (48.5%) and SSA (17%) were selected for this study to balance mechanical performance, sulfate resistance, and thermal shrinkage. On this basis, CSN was prepared as the control group without SSA or RA, CSR incorporated RA only, and CSRS incorporated both RA and SSA, as listed in Table 3. Each group included six parallel specimens. After preparation, all specimens were cured in a standard curing chamber at 20 °C and a relative humidity above 95%. Specimens were removed 1 day before testing and soaked in water for 24 h. The cylindrical specimens were used for unconfined compressive strength (UCS) and indirect tensile strength (ITS) tests, while the beam specimens were used for thermal shrinkage and sulfate resistance tests.

### 2.3. Experimental Program

#### 2.3.1. Unconfined Compressive Strength Test

**Figure 2:**
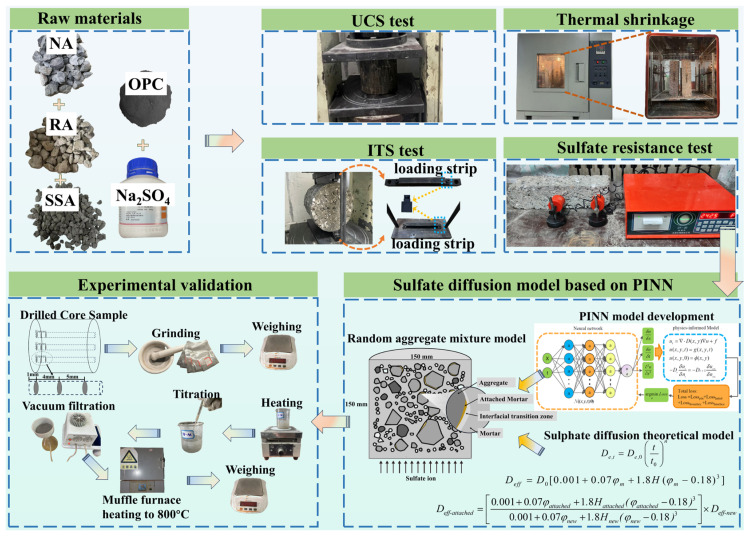
The flowchart of this study.

The UCS test was conducted in accordance with the Test Methods of Materials Stabilized with Inorganic Binders for Highway Engineering (JTG 3441-2024) [52]. A compression testing machine was used to apply loading at a rate of 1 mm/min, and the maximum load *P* at failure was recorded. The UCS was calculated using Equation (1). The experimental procedure used in this study is shown in Figure 2.

(1) $$R_{c}=\frac{P}{A}=\frac{4P}{\pi D^{2}}=0.000057P,$$

where *Rc* is the UCS of the specimen (MPa); *P* is the maximum load at failure (N); *A* is the cross-sectional area of the specimen (mm^2^); and *D* is the specimen diameter (mm).

#### 2.3.2. Indirect Tensile Strength Test

The ITS test was carried out in accordance with the Test Methods of Inorganic Binder Stabilized Materials for Highway Engineering. Before testing, the height of each water-immersed specimen was measured. Loading strips were then placed at the top and bottom of the specimen, and the loading rate of the compression testing machine was maintained at 1 mm/min. The maximum load *P* at failure was recorded. The test procedure is shown in Figure 2, and the ITS was calculated using Equation (2).

(2) $$R_{i}=\frac{2P}{\pi dh}(\sin2\alpha -\frac{a}{d})=0.004223\frac{P}{h},$$

where *R_i_* is the ITS of the specimen (MPa); *P* is the maximum load at failure (N); *a* is the width of the loading strip (mm); *α* is the central angle corresponding to half of the loading strip (rad); and *ℎ* is the height of the water-immersed specimen (mm).

#### 2.3.3. Thermal Shrinkage Test

The thermal shrinkage test was conducted using the strain-gauge method. Specimens at the required curing age were oven-dried to constant mass at 105 ± 1 °C. A strain gauge was attached along the central axis of each specimen. The temperature in the environmental chamber was first set to 40 °C and kept for 3 h to ensure the specimen reached the same temperature as the chamber. The temperature was then lowered in steps of 10 °C at a cooling rate of 0.5 °C/min, and each temperature level was maintained for 3 h. A resistance-type displacement transducer was used to measure the deformation. Cooling continued until the temperature reached −10 °C. The thermal shrinkage coefficient was calculated using Equation (3), and the test procedure is shown in Figure 2.

(3) $$\alpha_{i}=\frac{l_{i+1}-l_{i}}{L_{0}(t_{i+1}-t_{i})},$$

where *α_i_* is the thermal shrinkage coefficient; *l_i_* is the thermal shrinkage deformation at the *i*-th temperature (mm); *L*_0_ is the specimen length (mm); and *t_i_* is the temperature at the *i*-th temperature stage (°C).

#### 2.3.4. Sulfate Resistance Test

The sulfate attack test was conducted according to the Test Methods of Long-Term Performance and Durability of Concrete (GB/T 50082-2024) [54]. The resonance method was used to measure the dynamic elastic modulus of the specimens for evaluating their sulfate resistance. Beam specimens were cured under standard conditions for 28 days. Before sulfate exposure, the initial transverse fundamental frequency was measured using a DT-W18 dynamic modulus tester, and the initial dynamic elastic modulus *E*_0_ was calculated using Equation (4). After the initial measurements, the sides of each specimen were sealed with epoxy resin, and the bottom surface was immersed in a 10% Na_2_SO_4_ solution to accelerate sulfate attack and facilitate comparative evaluation of sulfate resistance. At the specified ages, the dynamic elastic modulus was measured again, and the sulfate resistance coefficient *K*_f_ was calculated using Equation (5).

(4) $$E=13.244\times 10^{-4}\times WL^{3}f^{2}/a^{4},$$

(5) $$K_{f}=\frac{E_{n}}{E_{0}}\times 100\%,$$

where *K*_f_ is the sulfate resistance coefficient (%), *W* is the mass of the specimen (kg), *L* and *a* are the specimen length and the side length of the square cross-section (mm), and *f* is the transverse fundamental frequencies of the specimen (Hz).

#### 2.3.5. Sulfate Diffusion Modeling Using PINN

1. PINN theoretical model;

PINN was applied to incorporate the chain rule of partial derivatives and physical constraint equations into the gradient backpropagation process through the loss function. The diffusion of sulfates in cement-stabilized materials was described by Fick’s second law of diffusion, which was expressed as a partial differential equation, as shown in Equation (6).

(6) $$\left\{\begin{array}{ll} \frac{\partial u_{t}}{\partial t}=D_{0}\cdot \left(\frac{\partial^{2}u}{\partial x^{2}}\right)+f, & x\in \Omega ,\\ f=-k\cdot u_{C_{3}A}\cdot u(x,t), & x\in \Omega ,\\ u(x,t)=g(x,t),t\in (0,T), & x\in \partial \Omega , \end{array}\right.$$

where *u*(*x*,*t*) is the sulfate ion concentration (mol/m^3^); *f* is the sulfate ion consumption due to chemical reactions; *D*_0_ is the diffusion coefficient (mm^2^/s); *g*(*x*,*t*) is the boundary condition under sulfate attack; Ω and *∂*Ω are the internal domain and boundary of specimens. *k* is the sulfate ion reaction rate coefficient, typically 3.05; *u_C_*_3*A*_ is the initial *C*_3_*A* concentration in concrete.

In this study, a feedforward neural network was used to approximate sulfate concentration with (*x*,*t*) as input variables, as shown in Equation (7).

(7) $$u_{\theta }=N((x,y,t);\theta ),$$

where *u*_θ_ is the sulfate ion concentration predicted by the neural network; N(·) is the feedforward neural network used in this study; and θ is the set of neural network parameters, including weights (*w*_θ_) and biases (bias_θ_).

The differential mechanism is used to obtain the first-order partial derivative $\partial u_{\theta }/\partial x$ and second-order partial derivative $\partial u_{\theta }/\partial t$ of the predicted value (*u*_θ_) for (*x*,*t*), which are then substituted into Equation (6). The residuals of the physical constraint equation, initial conditions, and boundary conditions were derived from the system of partial differential equations. In addition, abrupt changes in diffusion coefficients at interfaces due to material differences could introduce PINN prediction errors. To improve boundary accuracy, interface continuity conditions were incorporated into the loss function, ensuring smooth transport without additional losses. The interface continuity loss acted as a regularization term in the final neural network loss function, as shown in Equation (8).

(8) $$\begin{array}{l} \begin{array}{l} {\mathrm{Loss}}_{\mathrm{PDE}}=\frac{1}{N_{PDE}}\sum_{i=1}^{N_{PDE}}{\left|f\left(u_{\theta },\lambda ,x,t\right)\right|}^{2}\\ {\mathrm{Loss}}_{\mathrm{Initial}}=\frac{1}{N_{\mathrm{Initial}}}\sum_{i=1}^{N_{\mathrm{Initial}}}{\left|\mu_{\theta }(x,0)-g_{\mathrm{Initial}}\right|}^{2}\\ {\mathrm{Loss}}_{\mathrm{Boundary}\;}=\frac{1}{N_{\mathrm{Boundary}}}\sum_{i=1}^{N_{\mathrm{Boundary}}}{\left|\mu_{\theta }(x,t)-g_{\mathrm{Boundary}}\right|}^{2} \end{array}\\ {\mathrm{Loss}}_{Interface}=\frac{1}{N_{Interface}}\sum_{i=1}^{N_{Interface}}{\left|{\left.{\left.u_{i+1}\right|}_{x\in \Omega_{i+1}}-u_{i}\right|}_{x\in \Omega_{i}}\right|}^{2}\;\;\;\;\;\;\;\;\;\;\;\;\;\;\;\;\;\;\;\;\;\;\;\;\;\;\;\;\;\;\;\;,\\ \mathrm{Loss}=w_{1}{\mathrm{Loss}}_{\mathrm{PDE}}+w_{2}{\mathrm{Loss}}_{\mathrm{Initial}\;}+w_{3}{\mathrm{Loss}}_{\mathrm{Boundary}\;}+w_{4}{\mathrm{Loss}}_{\mathrm{Interface}\;} \end{array}$$

where Loss*_PDE_* is the partial differential equation loss of the system; Loss*_initial_*, Loss*_Boundary_*, and Loss*_Interface_* are the initial condition loss, boundary condition loss, and interface continuity loss, respectively. Loss is the final loss.

**Figure 3:**
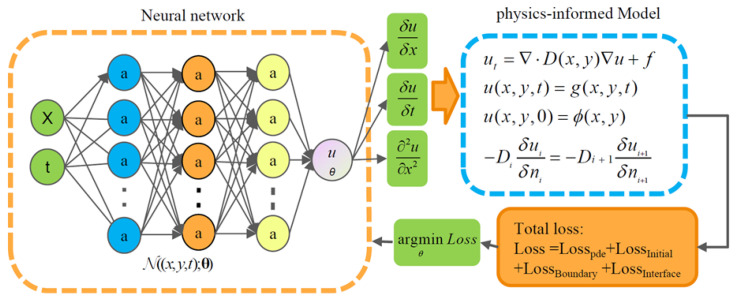
Schematic diagram of the PINN.

The sulfate diffusion in the cement-stabilized recycled aggregate systems was simulated using a physics-informed neural network (PINN). The network was a fully connected feedforward architecture with four hidden layers, each comprising 50 neurons, and employed the tanh activation function. Training was performed using the Adam optimizer with a learning rate of 1 × 10^−3^ for 15,000 epochs. Based on the principles of PINN and the underlying sulfate diffusion model, a PINN-based model specifically for sulfate diffusion in the cement-stabilized recycled aggregate systems was developed, as illustrated in Figure 3.

2. Sulfate diffusion coefficient;

The CSRS mixture contained multiple interfacial transition zones (ITZs) and mortar phases, including new mortar, attached mortar, and several ITZs: ITZ_1_ between NA and new mortar, ITZ_2_ between SSA and new mortar, ITZ_3_ between the attached mortar of RA and new mortar, ITZ_4_ between the original aggregate and attached mortar, and ITZ_5_ between the original aggregate and new mortar. To accurately characterize sulfate ion diffusion in the CSRS mixture, this study investigated the diffusion coefficients of each material phase.

- Sulfate diffusion coefficient in new mortar;

Hydration and sulfate attack in the mixture are continuous processes that gradually change the internal pore structure over time, making the sulfate diffusion rate time-dependent. This study applied the equation proposed by Min et al. to quantify the relationship between the effective diffusion coefficient and exposure time [55], as shown in Equation (9).

(9) $$D_{e,t}=D_{e,0}{\left(\frac{t}{t_{0}}\right)}^{n},$$

where *D_e,t_* is the effective diffusion coefficient of sulfate ions in new mortar (mm^2^/s); *D*_*e*,__0_ is the initial diffusion coefficient of sulfate ions in new mortar (mm^2^/s); *t*_0_ is the normalized time (60 days); *t* is the erosion age (days); and *n* is the diffusion attenuation coefficient.

According to Wu et al. [56], the time-dependent variation in the sulfate ion diffusion coefficient in the new mortar was calculated, as shown in Equation (10).

(10) $$D_{\text{eff-new}}=4.62\times {\left(\frac{60}{t}\right)}^{0.15}\times 10^{-7},$$

- Sulfate diffusion coefficient in attached mortar;

The attached mortar matrix has high porosity, which significantly influences the diffusion of sulfate ions. Following the approach of Wu et al. [56], the effective diffusion coefficient of the attached mortar was calculated based on porosity, as shown in Equation (11).

(11) $$D_{\mathrm{eff}}=D_{0}[0.001+0.07\varphi_{m}+1.8H{\left(\varphi_{m}-0.18\right)}^{3}],$$

where *D*_eff_ is the effective diffusion coefficient of sulfate ions in residual mortar (mm^2^/s); *D*_0_ is the diffusion coefficient of sulfate ions in aqueous solution (1.07 × 10^−9^ mm^2^/s); *φ*_m_ is the mortar porosity; and *H* is the Heaviside function, defined as *H* = 1 when *φ*_m_ > 0.18, and *H* = 0 otherwise.

The diffusion coefficient of sulfate ions in the attached mortar is correlated with that in the new mortar, as shown in Equation (12).

(12) $$D_{\text{eff-attached}}=\left[\frac{0.001+0.07\varphi_{\mathrm{attached}}+1.8H_{\mathrm{attached}}{\left(\varphi_{\mathrm{attached}}-0.18\right)}^{3}}{0.001+0.07\varphi_{\mathrm{new}}+1.8H_{\mathrm{new}}{\left(\varphi_{\mathrm{new}}-0.18\right)}^{3}}\right]\times D_{\text{eff-new}},$$

where *D*_eff-attached_ is the effective diffusion coefficient of sulfate ions in attached mortar (mm^2^/s); *φ*_attached_ is the porosity of attached mortar (%); *φ*_new_ is the porosity of new mortar (%); and *D*_eff-new_ is the effective diffusion coefficient of sulfate ions in new mortar (mm^2^/s).

- Sulfate diffusion coefficients in ITZs;

The sulfate ion effective diffusion coefficient in the ITZ was calculated using parameters summarized by Wu et al. [56]. For ITZ_2_, the interface between SSA and new mortar, the coefficient was determined based on the porosity measured by Chen et al. [23], as shown in Equation (13). According to the research by Xiao et al. [57], the ITZ thickness between new mortar and new aggregates is the same as that between new mortar and original aggregates, so the same parameters were used for ITZ_1_ and ITZ_5_. The sulfate ion effective diffusion coefficients for each ITZ phase in CSRS are listed in Table 4.

**Figure 4:**
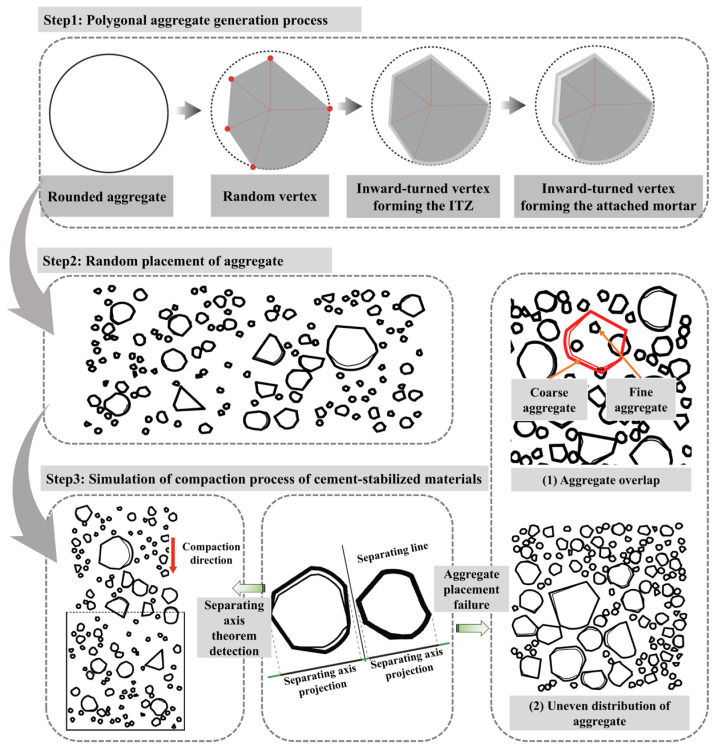
Generation of a random aggregate model based on compaction.

3. Random aggregate modeling;

The shape and distribution of aggregates play a significant role in the sulfate resistance of the mixture. In this study, irregular convex-edged aggregates were generated based on the properties of different aggregates. Furthermore, the aggregates were moved in the opposite direction of the expansion of the placement space to simulate the compaction process of pavement base mixtures under compaction pressure. To improve modeling density and generation efficiency, the separating axis theorem was applied to enhance the residual space method for distributing random aggregates, resulting in an efficient and precise random polygon aggregate model. The process for generating the random aggregate mixture model, based on the pavement base compaction, is shown in Figure 4.

**Figure 5:**
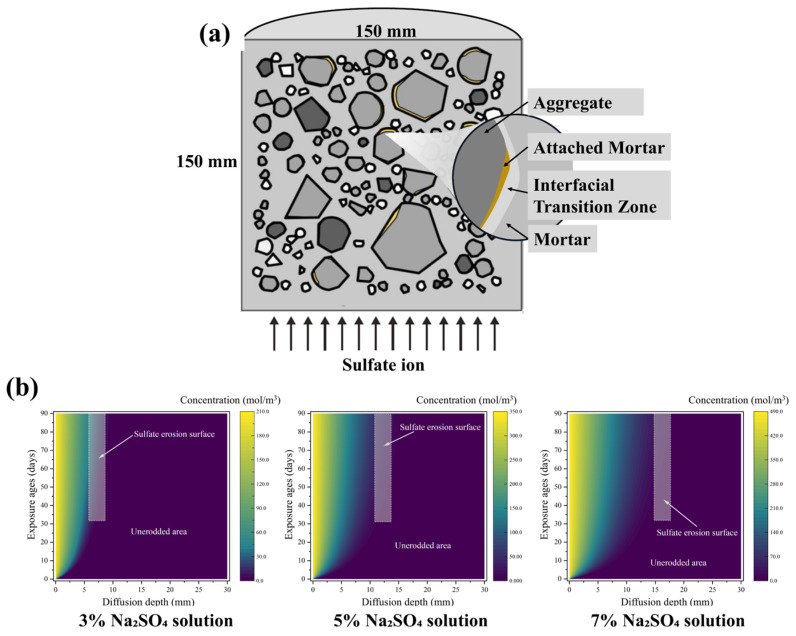
Sulfate diffusion behavior of mixtures: (**a**) Sulfate diffusion model for mixtures; (**b**) sulfate concentration distributions of CSR under different sulfate levels.

4. Sulfate diffusion model development;

The pavement base mixture model was designed as a cylindrical specimen with dimensions of 150 mm in height and 150 mm in diameter. The mixture proportions were determined according to Table 3. The initial sulfate concentration inside the specimen was set to 0 mol/m^3^, and the specimen was exposed to Na_2_SO_4_ solutions at concentrations of 3%, 5%, and 7% to simulate different sulfate attack conditions. Sulfate ingress was applied to the bottom surface, while the other surfaces were assumed to be impermeable with 0 sulfate flux (0 mol/m^2^). The boundary conditions were defined in Equation (13), and the generated diffusion model, along with the resulting concentration distributions, is presented in Figure 5.

(13) $$\left\{\begin{array}{l} c(x,y,0)=0,(x,y)\in \Omega \\ c(x,0,t)=c_{0},c(0,y,t)=0\\ \frac{\partial c_{x}(0,y,t)}{\partial n}=\frac{\partial c_{x}(x,y,t)}{\partial n} \end{array}\right.,$$

where Ω is the cross-sectional area of the specimen; $c_{x}(x,y,t)$ is the sulfate concentration inside the specimen (mol/m^3^); $\frac{\partial c_{x}(x,y,t)}{\partial n}$ is the normal sulfate flux on the side surface (mol/m^2^).

To assess the reliability of the sulfate diffusion model, the prepared specimens (CSN, CSR, and CSRS) were first cured under standard conditions for 28 days, followed by sealing the side surfaces with epoxy resin, leaving only the bottom surface exposed to the sulfate environment. After sealing, the specimens were immersed in a 5% sodium sulfate (Na_2_SO_4_) solution for 30, 60, and 90 days. Core samples were then extracted at depths of 1 mm, 5 mm, and 10 mm along the erosion direction. Following the method proposed by Wu et al. [56], the BaSO_4_ titration method was used to determine the sulfate ion concentration at different depths, providing an evaluation of sulfate attack on the specimens. The experimental procedure is shown in Figure 2.

## 3. Results

### 3.1. Unconfined Compressive Strength Analysis

**Figure 6:**
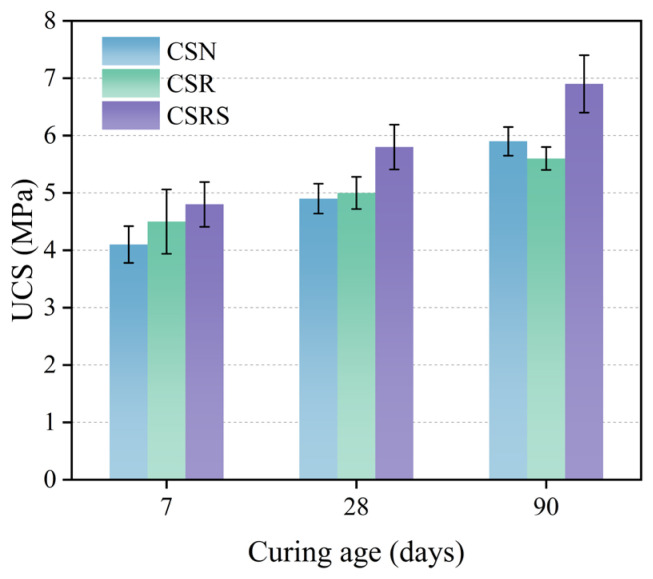
UCS of CSN, CSR, and CSRS at different curing ages.

Figure 6 presents the variations in UCS for CSN, CSR, and CSRS at curing ages of 7, 28, and 90 days. The UCS of all mixtures increased continuously with curing age, indicating ongoing hydration and progressive densification of the internal structure. A similar strength evolution trend was reported by Khan et al. [58], who found that cement-treated recycled aggregate achieved a UCS of 4.6 MPa at 7 days and 6.5 MPa at 28 days with 7% cement, confirming that the strength gain of recycled aggregate-based mixtures diminishes with prolonged curing. In addition, the incorporation of RA resulted in an early-age strength increase. The 7-day UCS of CSR was 9.8% higher than that of CSN. However, this beneficial effect weakened with curing age; at 28 days, the UCS was only 2.0% higher than CSN, and by 90 days, the UCS of CSR had fallen below that of CSN, reaching 94.9% of the CSN value. This trend is attributed to the rough residual mortar attached to the RA surface. At early ages, when the cement matrix remains relatively weak, the rough and porous surface of RA enhances mechanical interlocking and improves the bonding with the mortar. As hydration progresses, the residual mortar becomes the weak zone within the mixture, leading to reduced strength development at later ages.

The incorporation of SSA further improved the UCS of the mixtures at all curing ages. Compared with CSR, the UCS of CSRS increased by 6.7%, 16.0%, and 23.2% at 7, 28, and 90 days, respectively. This enhancement can be attributed to the incorporation of SSA, which provides additional calcium and iron-bearing phases (e.g., C_2_S, C_4_AF) that participate in secondary hydration reactions, producing more C–S–H and AFt gels.

Moreover, the angular morphology and higher surface roughness of SSA improved interfacial mechanical interlocking with the cementitious matrix, thereby strengthening the bonding performance between SSA and the mortar matrix interface. Consequently, CSRS exhibits a more compact and integrated microstructure, resulting in higher bearing capacity.

### 3.2. Indirect Tensile Strength Analysis

**Figure 7:**
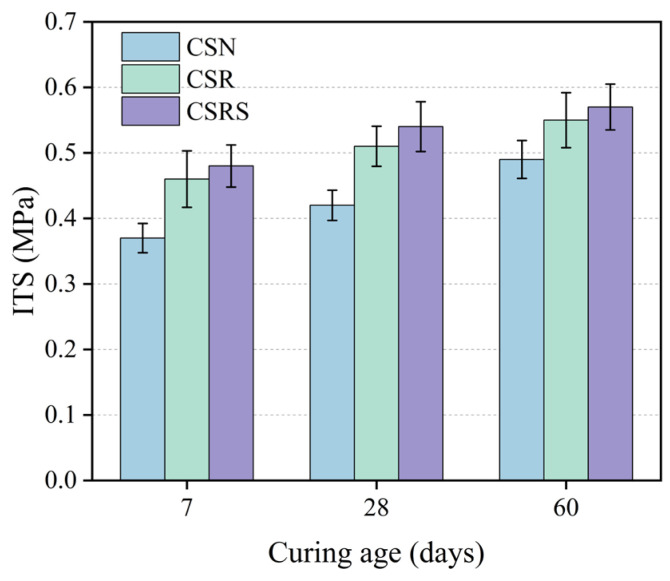
ITS of CSN, CSR, and CSRS at different curing ages.

Figure 7 shows the variations in ITS of CSN, CSR, and CSRS at curing ages of 7, 28, and 90 days. Compared with the 7-day ITS, the 28-day ITS of CSN, CSR, and CSRS increased by 13.5%, 10.9%, and 12.5%, respectively. From 28 to 90 days, the ITS further increased by 16.7%, 7.8%, and 5.6%. These results demonstrate a continuous strength gain with curing age due to ongoing hydration and the progressive densification of the mixture structure. With the incorporation of RA, the ITS of CSR increased by 24.3%, 21.4%, and 12.2% compared with CSN at 7, 28, and 90 days, respectively. CSRS showed additional increases of 4.3%, 5.9%, and 3.6% over CSR at the corresponding ages.

ITS is a key indicator of the resistance of pavement base materials to tensile failure and is mainly governed by the interlocking and bonding between aggregates and the cement mortar. The rough surface of RA and SSA allows the mortar to penetrate their surface pores, which enhances the bond between aggregates and mortar. Moreover, tensile cracks typically initiate and propagate along the ITZ around the aggregates. The incorporation of SSA improves the density and integrity of the ITZ, thereby contributing to the enhanced ITS of the pavement base.

### 3.3. Thermal Shrinkage Performance Analysis

**Figure 8:**
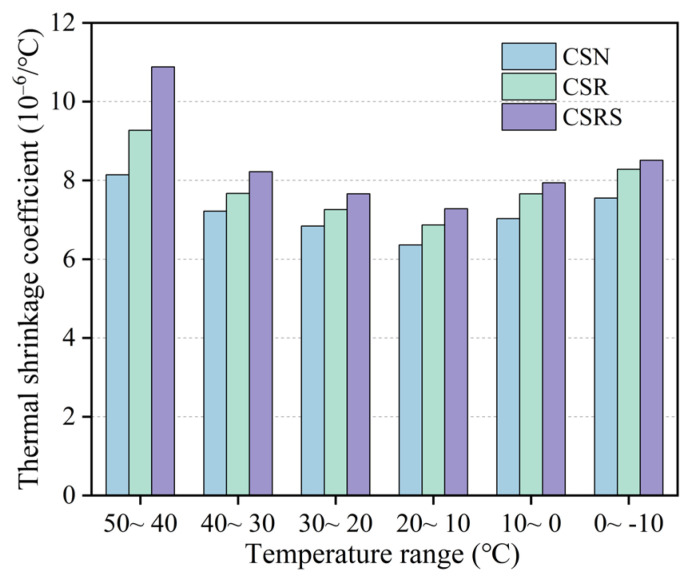
Thermal shrinkage coefficient of CSN, CSR, and CSRS at different temperature ranges.

Table 5 and Figure 8 show the variations in the thermal shrinkage coefficient of CSN, CSR, and CSRS within the temperature range of 50 °C to −10 °C. It can be observed that, under the same temperature conditions, the thermal shrinkage strain of CSR and CSRS is slightly higher than that of CSN. The average thermal shrinkage coefficients of CSN, CSR, and CSRS are 7.19 × 10^−6^/°C, 7.84 × 10^−6^/°C, and 8.42 × 10^−6^/°C, respectively. Compared with CSN, the average coefficients of CSR and CSRS increase by 9.0% and 17.1%, indicating that the incorporation of RA and SSA enhances the thermal shrinkage sensitivity of the mixtures. In the high-temperature interval (50~30 °C), the thermal shrinkage coefficient of CSRS was markedly higher than that of CSN, with a maximum increase exceeding 25%, suggesting that the higher thermal expansion coefficient and dense surface structure of SSA intensify the thermal deformation response. As the temperature decreased below 20 °C, the differences among the mixtures gradually diminished, and the thermal shrinkage tendencies became more uniform.

The higher thermal shrinkage coefficient of CSRS is mainly attributed to the intrinsic characteristics of SSA. Mineral phases such as C_2_S and MgO exhibit strong thermal sensitivity, and the dense internal structure of SSA limits its ability to relax thermal stresses. Moreover, the higher stiffness and stronger interfacial bonding restrict the release of thermal strain, resulting in greater cumulative shrinkage.

These findings indicate that while SSA incorporation enhances strength and crack resistance, it may also increase thermal shrinkage under temperature variations. In practical pavement base applications, this potential risk can be mitigated through measures such as fiber addition, optimized mixture design, and proper curing control, particularly in regions with large temperature fluctuations.

### 3.4. Sulfate Resistance Analysis

**Figure 9:**
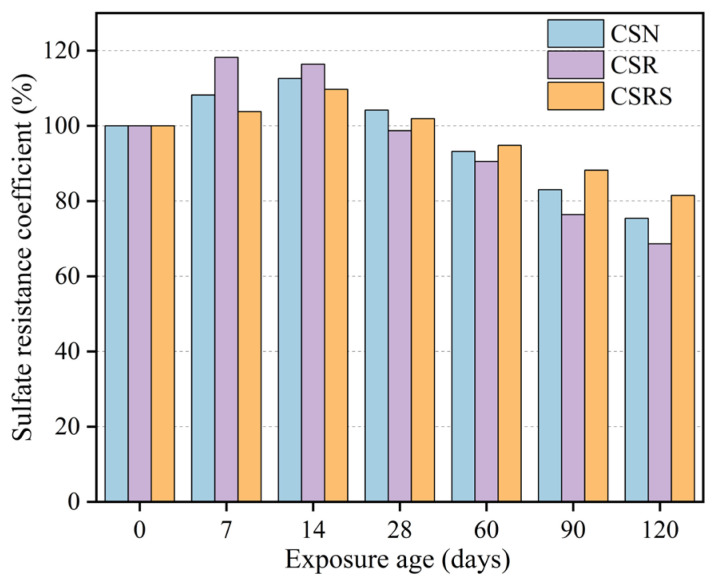
Sulfate resistance coefficients of CSN, CSR, and CSRS at different curing ages.

Figure 9 shows the variations in the sulfate resistance coefficient of CSN, CSR, and CSRS at different ages during exposure to a sodium sulfate solution. As illustrated in Figure 9, all mixtures exhibit an initial increase followed by a subsequent decline. CSR reached its maximum coefficient of 118.2% at 7 days, whereas CSN and CSRS reached their peak values of 112.6% and 109.7% at 14 days, respectively. With continued sulfate exposure, the coefficients decreased to 93.2%, 90.5%, and 94.8% at 60 days for CSN, CSR, and CSRS, respectively. After 120 days of exposure, the coefficients further declined to 75.4%, 68.6%, and 81.5%. Throughout the entire exposure period, CSRS consistently exhibited the highest sulfate resistance coefficient, indicating that the incorporation of SSA effectively improves the resistance of cement-stabilized mixtures to sulfate attack.

Based on the evolution and rate of change in the sulfate resistance coefficient, the sulfate attack process can be divided into three stages: the expansion densification stage, the accelerated deterioration stage, and the slow deterioration stage. In the expansion densification stage, sulfate ions react with C_3_A in the mixtures to produce expansive products such as ettringite (AFt) and gypsum. These products fill internal pores and microcracks, resulting in increased compactness and a corresponding rise in the resistance coefficient. Similar behavior was reported by Wang et al. [59], who found that during the initial sulfate exposure stage, the formation of ettringite contributed to pore filling and matrix densification, resulting in a mass variation within ±1% and an increase of approximately 10–15% in the sulfate resistance coefficient. This stage is relatively short and occurs mainly within the first 14 days. As the sulfate attack progresses, the mixtures enter the accelerated deterioration stage. Microcracks propagate rapidly, structural damage intensifies, and both the dynamic elastic modulus and resistance coefficient decline sharply. After approximately 60 days, the mixtures transition to the slow deterioration stage, during which microcrack development slows and the decrease in the resistance coefficient becomes more gradual.

Compared with CSN, CSR presents a more pronounced change in the resistance coefficient in both the expansion densification stage and the accelerated deterioration stage. This behavior can be attributed to the porous residual mortar and multiple interfacial transition zones within the recycled aggregate, which facilitate the penetration of sulfate ions and promote the formation of a larger volume of expansive products. The resulting damage develops more rapidly. In contrast, CSRS demonstrates consistently superior sulfate resistance throughout the entire exposure period. During the slow deterioration stage, its resistance coefficient is about 18.8% higher than that of CSR. This improvement is mainly due to the hydration of reactive mineral phases in SSA, which refines the pore structure, increases the density of the interfacial transition zone, and strengthens the bonding between aggregates and the cementitious matrix. These enhancements significantly hinder sulfate intrusion through weak interfacial regions and effectively slow the deterioration process, thereby improving the long-term durability of the mixture.

### 3.5. Prediction and Validation of the PINN-Based Sulfate Diffusion Model

**Figure 10:**
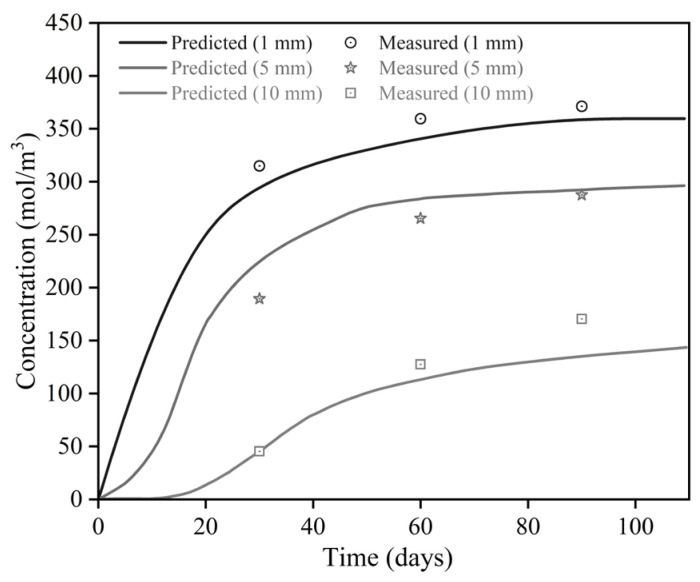
Comparison between experimental and simulated results at different exposure ages.

Figure 10 shows the comparison between simulated and experimental sulfate ion concentrations in CSR under 5% sulfate attack at different exposure periods. The results indicate that sulfate ion concentration increased with exposure time and then gradually stabilized, suggesting that sulfate attack primarily occurred in the early stages. As time progressed, the diffusion rate decreased, and the ion concentration approached dynamic equilibrium. The ion concentrations at 1 mm and 5 mm were relatively high, but a sharp decline occurred between 5 mm and 10 mm, with the measured concentration at 10 mm reaching only 44.84 mol/m^3^. This trend can be attributed to the formation of dense hydration products after 28 days, which progressively fill the pore structure, reduce pore connectivity, and hinder sulfate ion transport, resulting in a gradual decrease in concentration with depth.

The average relative errors between the experimental results and PINN model predictions at 1 mm, 5 mm, and 10 mm were 5.1%, 9.1%, and 10.7%, respectively. The maximum error of 20.7% occurred at 10 mm after 90 days. These deviations can be attributed to multiple factors, including experimental variability, sampling errors, the non-uniformity of material phases, and the simplified representation of ITZs in the model. Overall, these errors remain within an acceptable range and indicate that the PINN model reliably captures the sulfate diffusion behavior [60].

**Figure 11:**
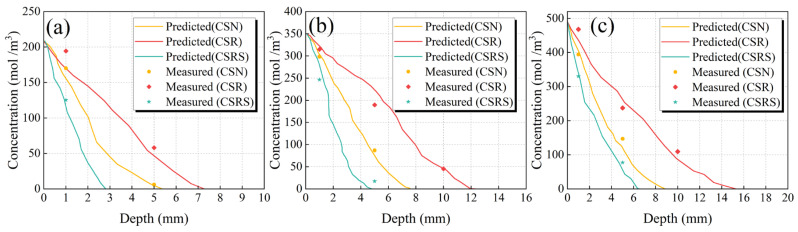
Comparison of experimental and simulated results at different sulfate concentrations: (**a**) 3%, (**b**) 5%, and (**c**) 7%.

The simulation results and experimental data of the mixture eroded at different sulfate concentrations for 30 days are shown in Figure 11. The results showed that the penetration depth increased with sulfate concentration but at a progressively slower rate. When the solution concentration increased from 3% to 5%, the maximum penetration depths of CSN, CSR, and CSRS samples increased by 40.6%, 63.7%, and 64.6%, respectively. However, when the concentration increased further from 5% to 7%, the increments slowed to 17.6%, 18.1%, and 37.2%, respectively. Additionally, at the same penetration depth, sulfate concentrations in CSRS samples remained lower than those in CSN and CSR. Notably, at 1 mm, the sulfate concentrations in CSRS were lower than those in CSN by 26.4%, 17.4%, and 16.2%, and lower than those in CSR by 35.6%, 21.8%, and 29.4%, under 3%, 5%, and 7% sulfate solutions, respectively. These results indicate that incorporating SSA effectively reduced sulfate ingress and significantly enhanced the sulfate resistance of CSR.

The average relative errors between the experimental results and PINN model predictions were 13.9%, 8.3%, and 12.8% for sulfate concentrations of 3%, 5%, and 7%, respectively. All errors fell within an acceptable range. These results indicate that the PINN model exhibited favorable predictive accuracy and reliability, demonstrating its effectiveness for simulating sulfate attack in pavement base mixtures.

## 4. Conclusions

This study investigated the effectiveness of incorporating SSA into CSR mixtures to enhance their mechanical performance and resistance to sulfate attack. Experimental tests on UCS, ITS, thermal shrinkage behavior, and sulfate resistance were conducted, and a PINN model was established to predict sulfate diffusion behavior. The main conclusions are as follows.

(1) The incorporation of SSA significantly improved the mechanical properties of CSR. Compared with CSR, the UCS of CSRS at 7, 28, and 90 days increased by 6.7%, 16.0%, and 23.2%, respectively. For ITS, CSRS exhibited increases of 4.3%, 5.9%, and 3.6% at 7, 28, and 60 days, respectively. These enhancements were mainly attributed to the hydration of reactive mineral phases in SSA and its rough surface texture, which strengthened the interfacial bonding between aggregates and mortar and consequently improved the overall mechanical performance of the mixture.
(2) The incorporation of RA and SSA increased the thermal shrinkage sensitivity of the mixtures. The average thermal shrinkage coefficients of CSR and CSRS increased by 9.0% and 17.1% relative to CSN. This finding highlights a potential risk associated with temperature-induced deformation, particularly in regions with large temperature fluctuations. Therefore, appropriate control of SSA content and the adoption of shrinkage mitigation measures should be considered in practical applications.
(3) SSA significantly enhanced the sulfate resistance of CSR. Compared with CSR, the sulfate resistance coefficient of CSRS at 120 days increased by 18.8%. The weaker performance of CSR was associated with the greater amount of internal defects and pores in RA, which accelerated deterioration under prolonged sulfate exposure. The incorporation of SSA reduced these defects, improved the compactness of the mixture, and slowed the progression of sulfate attack, thereby enhancing the long-term durability of the pavement base.
(4) A novel sulfate diffusion model for pavement base mixtures was developed based on PINN, and the results showed that the incorporation of SSA effectively reduced sulfate ingress, with sulfate concentrations in CSRS samples at 1 mm lower than those in CSR by 35.6%, 21.8%, and 29.4%, respectively, under 3%, 5%, and 7% sulfate solutions. The PINN model exhibited reliable predictive accuracy, with average relative errors of 13.9%, 8.3%, and 12.8% for 3%, 5%, and 7% sulfate solutions. This confirms the feasibility of the proposed PINN framework for simulating sulfate transport in cement-stabilized mixtures and supporting durability assessment.

This study confirms that incorporating SSA enhances the mechanical performance and sulfate resistance of CSR and that the proposed PINN model provides a feasible approach for predicting sulfate diffusion behavior. As the present model focuses solely on sulfate diffusion, future research should investigate the coupled effects of cement type and content, sulfate diffusion, and the evolution of mechanical indicators such as dynamic modulus and sulfate resistance coefficients, to establish a more comprehensive durability evaluation framework for cement-stabilized base materials.

## Figures and Tables

**Table 1:** Basic properties of ordinary Portland cement.

Test Items		Test Results	Technical Requirements
Specific surface area (m^2^/kg)		343	≥300
80 μm square-hole sieve, weigh to residue (%)		2.3	≤10
Setting time (min)	Initial setting	235	≥180
	Final setting	425	≤600
Compressive strength (MPa)	3d	29.7	≥17.0
	28d	58.4	≥42.5
Flexural strength (MPa)	3d	5.2	≥4.0
	28d	7.9	≥7.0

**Table 2:** Basic properties of aggregates.

Test Items	Crushing Value	Needle-Like Particle Content	Apparent Density (g/m^3^)	Water Absorption (%)	Dust Content Below 0.075 mm (%)	Sulfate Content (%)	Water Immersion Expansion Rate (%)
RA (5–10 mm)	23	7.5	2.435	4.35	0.8	0.2	-
RA (10–20 mm)	23	7.5	2.467	4.42	0.8	0.2	-
SSA (5–10 mm)	13.4	0.8	3.282	1.93	-	-	1.76
SSA (10–20 mm)	13.4	0.8	3.341	1.88	-	-	1.76
NA (5–30 mm)	17.3	11.2	2.68	1.26	-	-	-
NA (0–5 mm)	-	-	2.63	2.45	-	-	-
Technical Requirements	≤30	≤18	≥2.35	≤5.0	≤1.2	≤0.25	≤2.0

**Table 3:** Mix proportions of cement-stabilized natural aggregate, cement-stabilized recycled aggregate, and cement-stabilized recycled aggregate with steel slag.

Experimental Program	OPC Content (%)	RA (%)	SSA (%)	Optimum Moisture Content (%)	Maximum Dry Density (g/cm^3^)
CSN	4.5	0	0	3.9	2.394
CSR	4.5	48.5	0	5.0	2.276
CSRS	4.5	48.5	17	5.3	2.378

**Table 4:** Sulfate diffusion coefficient in different interfacial transition zones.

Aggregate Types	ITZ	Porosity (%)	Effective Diffusion Coefficient
NA	ITZ_1_	27.0	9.48 *D*_eff-new_
SSA	ITZ_2_	14.24	0.88 *D*_eff-new_
RA	ITZ_3_	22.5	2.05 *D*_eff-new_
	ITZ_4_	33.75	1.25 *D*_eff-new_
	ITZ_5_	27.0	9.48 *D*_eff-new_

**Table 5:** Test results of thermal shrinkage strain and thermal shrinkage coefficient.

Temperature Range (°C)	Cumulative Thermal Strain (×10^−6^)			Thermal Shrinkage Coefficient (×10^−6^/°C)		
	CSN	CSR	CSRS	CSN	CSR	CSRS
50~40	81.4	92.7	108.8	8.14	9.27	10.88
40~30	162.8	185.4	217.6	7.22	7.67	8.22
30~20	235.0	262.1	299.8	6.84	7.26	7.66
20~10	303.4	334.7	376.4	6.36	6.87	7.28
10~0	367	403.4	449.2	7.03	7.66	7.94
0~−10	437.3	480	528.6	7.55	8.28	8.51
Average				7.19	7.84	8.42

## Data Availability

The original contributions presented in the study are included in the article, further inquiries can be directed to the corresponding author.

## Abbreviations

The following abbreviations are used in this manuscript:

RA	Recycled aggregate
NA	Natural aggregate
SSA	Steel slag aggregate
CSR	Cement-stabilized recycled aggregate
CSN	Cement-stabilized natural aggregate
CSRS	Cement-stabilized recycled aggregate with steel slag
CSS	Cement-stabilized steel slag
PINN	Physics-informed neural network
OPC	Ordinary Portland cement
UCS	Unconfined compressive strength
ITS	Indirect tensile strength
C&D	Construction and demolition
ITZ	Interfacial transition zone
AFt	Ettringite

## References

[1] Akhtar A., Sarmah A.K. (2018). Construction and Demolition Waste Generation and Properties of Recycled Aggregate Concrete: A Global Perspective. J. Clean. Prod..

[2] Nedeljković M., Visser J., Šavija B., Valcke S., Schlangen E. (2021). Use of Fine Recycled Concrete Aggregates in Concrete: A Critical Review. J. Build. Eng..

[3] Zeng Q., Jike N., Xu C., Yang R., Peng Y., Wang J., Gong F., Zhang M., Zhao Y. (2022). Total Recycling of Low-Quality Urban-Fringe Construction and Demolition Waste towards the Development of Sustainable Cement-Free Pervious Concrete: The Proof of Concept. J. Clean. Prod..

[4] Gálvez-Martos J.-L., Styles D., Schoenberger H., Zeschmar-Lahl B. (2018). Construction and Demolition Waste Best Management Practice in Europe. Resour. Conserv. Recycl..

[5] Chakravarthi S., Shankar S. (2021). Utilization of Recycled Aggregates in Cement-Treated Bases: A State-of-the-Art Review. Innov. Infrastruct. Solut..

[6] Yuan Y., Hu X., Wang K., Liu Z., Zhong M., Meng K. (2024). Study on Mechanical Properties of Road Cement-Stabilized Macadam Base Material Prepared with Construction Waste Recycled Aggregate. Buildings.

[7] Joseph H.S., Pachiappan T., Avudaiappan S., Maureira-Carsalade N., Roco-Videla Á., Guindos P., Parra P.F. (2023). A Comprehensive Review on Recycling of Construction Demolition Waste in Concrete. Sustainability.

[8] Zega C.J., Santillán L.R., Sosa M.E., Villagrán Zaccardi Y.A. (2020). Durable Performance of Recycled Aggregate Concrete in Aggressive Environments. J. Mater. Civ. Eng..

[9] Shi X., Mukhopadhyay A., Zollinger D. (2019). Long-Term Performance Evaluation of Concrete Pavements Containing Recycled Concrete Aggregate in Oklahoma. Transp. Res. Rec..

[10] Sadati S., Khayat K.H. (2016). Field Performance of Concrete Pavement Incorporating Recycled Concrete Aggregate. Constr. Build. Mater..

[11] Medina C., Zhu W., Howind T., Sánchez de Rojas M.I., Frías M. (2014). Influence of Mixed Recycled Aggregate on the Physical—Mechanical Properties of Recycled Concrete. J. Clean. Prod..

[12] Fan M., Lyu Z., Liu L., Qin J., Liang G., Huang N. (2024). Preparation of Pavement Base Material by Using Steel Slag Powder and Steel Slag Aggregate. Matéria.

[13] Liu J., Yu B., Wang Q. (2020). Application of Steel Slag in Cement Treated Aggregate Base Course. J. Clean. Prod..

[14] Aiban S. (2006). Utilization of Steel Slag Aggregate for Road Bases. J. Test. Eval..

[15] Guo Y., Zhang W., Su H., Li J., Xu P., Wang J., Tian B. (2025). Optimal Selection of Chemical Composition of Pavement Materials Based on Wave-Absorbing Heating Characteristics and Design of de-Icing Functional Layer for Pavements. Case Stud. Constr. Mater..

[16] Liu Q., Xue G., Dong W., Li J. (2025). Compressive Behavior of Steel Slag Fine Aggregate Concrete under Impact Loading. Struct. Concr..

[17] Wang Z., Feng Z., Cui Q., Li X., Zhang L., Yang X., Li X. (2025). Crack Monitoring and Self-Healing Properties of Multifunctional Asphalt Mixture Containing Steel Slag and Carbon Fiber towards Sustainable Pavement. Sustain. Mater. Technol..

[18] Kumar P., Shukla S. (2023). Utilization of Steel Slag Waste as Construction Material: A Review. Mater. Today Proc..

[19] Ren Z., Li D., Ren Z., Li D. (2023). Application of Steel Slag as an Aggregate in Concrete Production: A Review. Materials.

[20] Li Y., Liu F., Yu F., Du T. (2024). A Review of the Application of Steel Slag in Concrete. Structures.

[21] Cai Y.-H., Huang T., Huang B.-Y., Hua C.-B., Huang Q., Chen J.-W., Liu H.-L., He Z.-J., Rouzi N.-B., Xie Z.-H. (2025). Investigation into the Static Mechanical Properties of Ultra-High-Performance Geopolymer Concrete Incorporating Steel Slag, Ground Granulated Blast-Furnace Slag, and Fly Ash. Buildings.

[22] Zhao Y., Shi T., Cao L., Kan L., Wu M. (2021). Influence of Steel Slag on the Properties of Alkali-Activated Fly Ash and Blast-Furnace Slag Based Fiber Reinforced Composites. Cem. Concr. Compos..

[23] Chen G., Wang S. (2023). Research on Macro-Microscopic Mechanical Evolution Mechanism of Cement-Stabilized Steel Slag. J. Build. Eng..

[24] Tozsin G., Yonar F., Yucel O., Dikbas A. (2023). Utilization Possibilities of Steel Slag as Backfill Material in Coastal Structures. Sci. Rep..

[25] Song P.-C., Chen G.-X., Chen Y.-J., Song P.-C., Chen G.-X., Chen Y.-J. (2024). Optimizing the Utilization of Steel Slag in Cement-Stabilized Base Layers: Insights from Freeze–Thaw and Fatigue Testing. Materials.

[26] Huang Y., Yang X., Wang S., Liu Z., Liu L., Xu B., Huang Y., Yang X., Wang S., Liu Z. (2022). Evaluating Cement Treated Aggregate Base Containing Steel Slag: Mechanical Properties, Volume Stability and Environmental Impacts. Materials.

[27] Soliman N., Roghani H., Aghayan I., Omran A., Sobolev K. (2025). Utilization of Steelmaking By-Products in the Construction Industry: A Comprehensive Review of Steel Slag and Steel Mill Scale. Case Stud. Constr. Mater..

[28] Zhang J., Su P., Li L. (2023). Microbial Induced Carbonate Precipitation Modified Steel Slag: Mechanical Improvement and Erosion Resistance to Sulfate Attack. J. Clean. Prod..

[29] Cheng X., Tian W., Gao J., Gao Y. (2022). Performance Evaluation and Lifetime Prediction of Steel Slag Coarse Aggregate Concrete under Sulfate Attack. Constr. Build. Mater..

[30] Vo D.-H., Do N.-D., Mamuye Y., Liao M.-C., Hwang C.-L., Tran Q.-T. (2022). Engineering Properties and Durability of Concrete Samples Designed by Densified Mixture Design Algorithm (DMDA) Method Incorporating Steel Reducing Slag Aggregate. Constr. Build. Mater..

[31] Feng T., Wang W., Li N., Luo J., Li B., Jiang P., Pu S. (2024). Mechanical Properties and Microscopic Mechanism of Steel Slag, Sodium Sulfate and Cement Stabilized Road Demolition Waste. Results Eng..

[32] O’Connor J., Nguyen T.B.T., Honeyands T., Monaghan B., O’Dea D., Rinklebe J., Vinu A., Hoang S.A., Singh G., Kirkham M.B. (2021). Production, Characterisation, Utilisation, and Beneficial Soil Application of Steel Slag: A Review. J. Hazard. Mater..

[33] Baras A., Li J., Ni W., Hussain Z., Hitch M. (2023). Evaluation of Potential Factors Affecting Steel Slag Carbonation. Processes.

[34] Guo J., Bao Y., Wang M. (2018). Steel Slag in China: Treatment, Recycling, and Management. Waste Manag..

[35] Zhang X., Li H., Li S., Ding Y., Zhang H., Tong Y., Hua S. (2022). Test and Microstructural Analysis of a Steel Slag Cement-Based Material Using the Response Surface Method. Materials.

[36] Deng Y., Wang X., Zhou B., Xu X., Chen L. (2024). Systematic Assessment of a Multi-Solid Waste Cementitious Material: Feasibility and Environmental Impact. Constr. Build. Mater..

[37] Sharba A.A. (2019). The Efficiency of Steel Slag and Recycled Concrete Aggregate on the Strength Properties of Concrete. KSCE J. Civ. Eng..

[38] Qin S., Chen C., Zhang M., Qin S., Chen C., Zhang M. (2024). Modeling of Concrete Deterioration under External Sulfate Attack and Drying–Wetting Cycles: A Review. Materials.

[39] Wu H., Lv C., Xu Y., Sun Y., Qu S., Zhou X., Wu H., Lv C., Xu Y., Sun Y. (2025). Deterioration of Concrete Under the Combined Action of Sulfate Attack and Freeze–Thaw Cycles: A Review. Materials.

[40] Zhuang Z., Mu S., Guo Z., Liu G., Zhang J., Miao C. (2024). Diffusion-Reaction Models for Concrete Exposed to Chloride-Sulfate Attack Based on Porosity and Water Saturation. Cem. Concr. Compos..

[41] Zhang T., Wang D., Lu Y. (2023). RheologyNet: A Physics-Informed Neural Network Solution to Evaluate the Thixotropic Properties of Cementitious Materials. Cem. Concr. Res..

[42] Gao Z., Fu Z., Wen M., Guo Y., Zhang Y. (2024). Physical Informed Neural Network for Thermo-Hydral Analysis of Fire-Loaded Concrete. Eng. Anal. Bound. Elem..

[43] Pasupunuri S.K., Thom N., Li L. (2024). Roughness Prediction of Jointed Plain Concrete Pavement Using Physics Informed Neural Networks. Transp. Res. Rec..

[44] Rahman M.A., Lu Y. (2024). EcoBlendNet: A Physics-Informed Neural Network for Optimizing Supplementary Material Replacement to Reduce the Carbon Footprint during Cement Hydration. J. Clean. Prod..

[45] Shaban W.M., Elbaz K., Zhou A., Shen S.-L. (2023). Physics-Informed Deep Neural Network for Modeling the Chloride Diffusion in Concrete. Eng. Appl. Artif. Intell..

[46] (2020). Testing Methods of Cement and Concrete for Highway Engineering.

[47] (2021). Technical Specifications for Utilization of Construction Waste in Highway Engineering.

[48] (2009). Test Method for Stability of Steel Slag.

[49] (2010). Steel Slag for Road.

[50] (2024). Test Methods of Aggregates for Highway Engineering.

[51] (2015). Technical Guidelines for Construction of Highway Roadbases.

[52] (2024). Test Methods of Materials Stabilized with Inorganic Binders for Highway Engineering.

[53] Deng Y., Wang X., Zhao J., Liu H., Chen L. (2025). Enhancing Sulfate Resistance of Cement-Stabilized Recycled Aggregate with Steel Slag: Optimized Mix Design and Mechanistic Insights. Case Stud. Constr. Mater..

[54] (2024). Standard for Test Methods of Long-Term Performance and Durability of Concrete.

[55] Min H., Sui L., Xing F., Tian H., Zhou Y. (2019). An Effective Transport Model of Sulfate Attack in Concrete. Constr. Build. Mater..

[56] Wu J., Wen X., Guan B., Yin G., Qiu H., Zhao H., Li C. (2023). Numerical Investigation of Sulfate Diffusion Characteristics in Recycled Aggregate Concrete Based on Mesoscale Multiphase Analysis. J. Mater. Civ. Eng..

[57] Xiao J., Li W., Sun Z., Lange D.A., Shah S.P. (2013). Properties of Interfacial Transition Zones in Recycled Aggregate Concrete Tested by Nanoindentation. Cem. Concr. Compos..

[58] Khan Z.A., Balunaini U., Nguyen N.H.T., Costa S. (2024). Evaluation of Cement-Treated Recycled Concrete Aggregates for Sustainable Pavement Base/Subbase Construction. Constr. Build. Mater..

[59] Wang Q., Liu J., Wang P., Liu J., Sun M., Wang Q., Liu J., Wang P., Liu J., Sun M. (2024). Effect of Sulfate Attack on the Expansion Behavior of Cement-Treated Aggregates. Materials.

[60] Caré S. (2003). Influence of Aggregates on Chloride Diffusion Coefficient into Mortar. Cem. Concr. Res..

